# Brain MRI in SARS-CoV-2 pneumonia patients with newly developed neurological manifestations suggestive of brain involvement

**DOI:** 10.1038/s41598-021-00064-5

**Published:** 2021-10-14

**Authors:** Batil Alonazi, Ahmed M. Farghaly, Mohamed A. Mostafa, Jehad A. Al-Watban, Salah A. Zindani, Feras Altaimi, Moram A. Fagiry, Mustafa Z. Mahmoud

**Affiliations:** 1 Radiology and Medical Imaging Department, College of Applied Medical Sciences, Prince Sattam Bin Abdulaziz University, PO Box 422, Al-Kharj, 11942 Saudi Arabia; 2 Medical Imaging Department, Prince Mohammed Bin Abdulaziz Hospital, Riyadh, Saudi Arabia; 3 Diagnostic Radiologic Technology Department, College of Medical Radiological Sciences, Sudan University of Science and Technology, Khartoum, Sudan

**Keywords:** Diseases, Medical research

## Abstract

The increased frequency of neurological manifestations, including central nervous system (CNS) manifestations, in patients with coronavirus disease 2019 (COVID-19) pandemic is consistent with the virus's neurotropic nature. In most patients, brain magnetic resonance imaging (MRI) is a sensitive imaging modality in the diagnosis of viral encephalitides in the brain. The purpose of this study was to determine the frequency of brain lesion patterns on brain MRI in severe acute respiratory syndrome coronavirus 2 (SARS-CoV-2) pneumonia patients who developed focal and non-focal neurological manifestations. In addition, it will compare the impact of the Glasgow Coma Scale (GCS) as an index of deteriorating cerebral function on positive brain MRIs in both neurological manifestations. This retrospective study included an examination of SARS-CoV-2 pneumonia patients with real-time reverse transcription polymerase chain reaction (RT-PCR) confirmation, admitted with clinicoradiologic evidence of COVID-19 pneumonia, and who were candidates for brain MRI due to neurological manifestations suggesting brain involvement. Brain imaging acquired on a 3.0 T MRI system (Skyra; Siemens, Erlangen, Germany) with a 20-channel receive head coil. Brain MRI revealed lesions in 38 (82.6%) of the total 46 patients for analysis and was negative in the remaining eight (17.4%) of all finally enclosed patients with RT-PCR confirmed SARS-CoV-2 pneumonia. Twenty-nine (63%) patients had focal neurological manifestations, while the remaining 17 (37%) patients had non-focal neurological manifestations. The patients had a highly significant difference (*p* = 0.0006) in GCS, but no significant difference (*p* = 0.4) in the number of comorbidities they had. Brain MRI is a feasible and important imaging modality in patients with SARS-CoV-2 pneumonia who develop neurological manifestations suggestive of brain involvement, particularly in patients with non-focal manifestations and a decline in GCS.

## Introduction

A variable percentage of patients with real-time reverse transcription polymerase chain reaction (RT-PCR) confirmed severe acute respiratory syndrome coronavirus 2 (SARS-CoV-2) pneumonia had neurological symptoms. When such manifestations were suggestive of central nervous system (CNS) involvement, brain cross-sectional imaging was recommended^1–3^. During the first month of admission, a relatively high percentage (36%) of CNS symptoms, including headache, altered mental status, acute cerebrovascular disease manifestations (e.g., weakness, aphasia), and epilepsy, were reported in patients with SARS-CoV-2 pneumonia^4^. A common clinical neurological manifestation of SARS-CoV-2 pneumonia is altered mental status or impaired sensorium, which can be attributed to respiratory distress and hypoxia. This necessitates neuroimaging^5^. Furthermore, neurologic symptoms are more common in coronavirus disease 2019 (COVID-19) pandemic patients who have a more severe respiratory disease status^4^. Anecdotal evidence suggests that patients with COVID-19 are predisposed to ischemic and hemorrhagic CNS complications. In most cases, brain magnetic resonance imaging (MRI) is a sensitive imaging modality in the diagnosis of viral encephalitides in the brain^6^.

The aim of this research was to assess the frequency of brain lesion patterns on brain MRI in SARS-CoV-2 pneumonia patients who developed focal and non-focal neurological symptoms. In addition, it will compare the impact of Glasgow Coma Scale (GCS) as an index of deteriorating cerebral function on positive brain MRIs in both neurological manifestations.

## Material and methods

### Study design and patient selection

**Figure 1:**
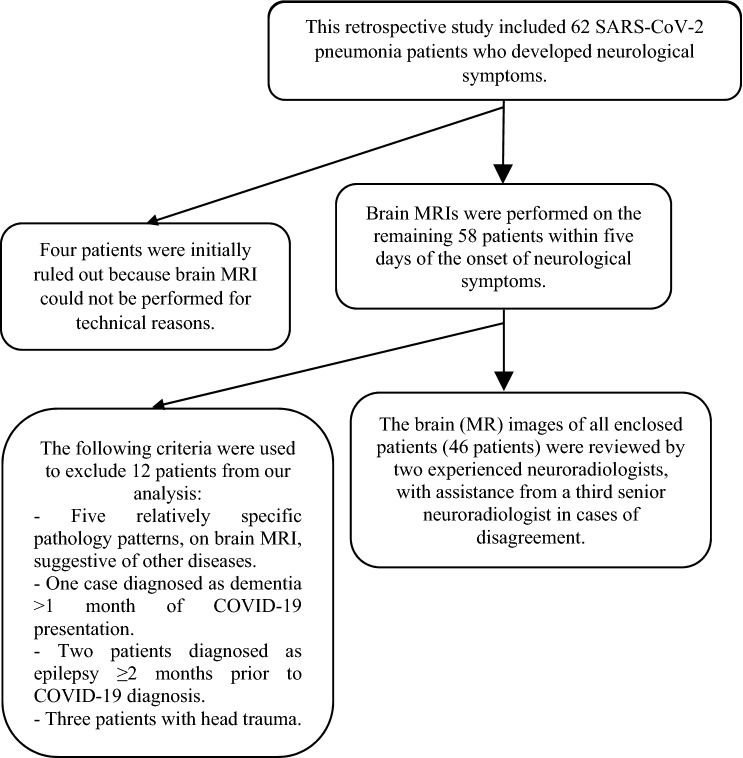
Depicts the descriptive selection of RT-PCR confirmed SARS-CoV-2 pneumonia patients, presented with neurological manifestations and subjected to brain MRI, that are enclosed in this study analysis.

This retrospective study comprised an analysis of 62 RT-PCR confirmed SARS-CoV-2 pneumonia patients, admitted at Prince Mohammed bin Abdulaziz Hospital between 15 May 2020 and 15 July 2020 with clinicoradiologic evidence of COVID-19 pneumonia, who were candidates for brain MRI, as they developed neurological manifestations suggesting brain involvement. The Research Ethics Board Committee of Prince Mohammed bin Abdulaziz Hospital approved this study. Patients were enrolled after providing written and verbal informed consent. Prior to the start of the study, informed consent was obtained from all patients and/or their legal guardians (for participants under the age of 18) for both study participation and publication of identifying information/images in an online open-access publication. The ethics procedures used were in accordance with the 1975 Helsinki Declaration, which was updated in 2013. Four patients were initially excluded, as brain MRI could not be performed, due to technical reasons (Fig. 1).

### Brain MRI technique and equipment in SARS-CoV-2 pneumonia patients

Brain MRI was performed, within five days from the onset of developing the neurological manifestations, for the remaining 58 patients, as per the following protocol: Brain imaging acquisitions were performed for every patient on a 3.0 T MRI system (Skyra; Siemens, Erlangen, Germany) with a 20-channel receive head coil. The protocol included T_1_-weighted (WI), T_2_-WI turbo spin-echo, fluid-attenuated inversion recovery (FLAIR), diffusion-weighted MRI (DW-MRI), and gradient-echo T_2_-WI imaging. Gadolinium-diethylenetriamine pentaacetic acid (Gd-DTPA) intravenous (IV) contrast injection was administered in 28 patients (60.9%). Post-contrast sequences included axial, coronal, and sagittal spin-echo post-contrast images of 3 mm thickness. The brain MRI scan was performed in accordance with the Kremer et al.^7^ guidelines on COVID-19 infection and magnetic resonance (MR) use. Three-dimensional (3D) T_1_-WI spin-echo MRI with or without contrast enhancement, diffusion-weighted imaging (DWI), gradient-echo T_2_- or susceptibility-WI, and two- or three-dimensional (2D or 3D) FLAIR imaging after administration of a gadolinium-based contrast agent were the sequences used by Kremer et al.^7^

All enclosed patients’ brain (MR) images were reviewed by two experienced neuroradiologists, with the help of a third senior neuroradiologist in cases of disagreement. Twelve patients were then excluded from our analysis due to the following criteria (Fig. 1): (1) Five relatively specific pathology patterns, on brain MRI, suggestive of other diseases: multiple sclerosis: four patients and meningeal brain tumor: one patient each. (2) One patient diagnosed as dementia > 1 month of COVID-19 presentation. (3) Two patients diagnosed as epilepsy ≥ 2 months prior to COVID-19 diagnosis. (4) Three patients with head trauma.

The clinicolaboratory data for the symptoms, clinical examination, laboratory data, chest radiogram, and computed tomography (CT) scanning were thoroughly reviewed. All enclosed patients showed positive RT-SARS-CoV-2 analysis of nasopharyngeal swabs and variably extended characteristic patterns as ground-glass opacities (GGOs), sublobar infiltrates, and/or crazy-paving pattern on chest CT scanning. Seven comorbidities were verified in each patient who was given a score (1 for each comorbidity for quantitative analysis). Those comorbidities were hypothyroidism, hypertension (HTN), diabetes mellitus (DM), ischemic heart disease (IHD), heart failure (HF), end stage renal disease (ESRD), malignancy (solid or hematologic).

### Statistical analysis

A descriptive analysis was performed on the various above-mentioned brain lesion patterns on brain MRI in SARS-CoV-2 pneumonia patients who developed focal and non-focal neurological manifestations, as well as their mean global percentage affecting the CNS. Statistical Package for the Social Sciences (SPSS) version 20 for Windows (IBM Corporation, Armonk, NY, USA) was used to perform descriptive analyses and calculate the means and standard deviations (SD). Pearson’s and Spearman’s correlations were used to assess the relationships between the two variables. The significance of the findings was determined using the *p* value. A *p* ≤ 0.05 value was considered significant.

## Results

**Figure 2:**
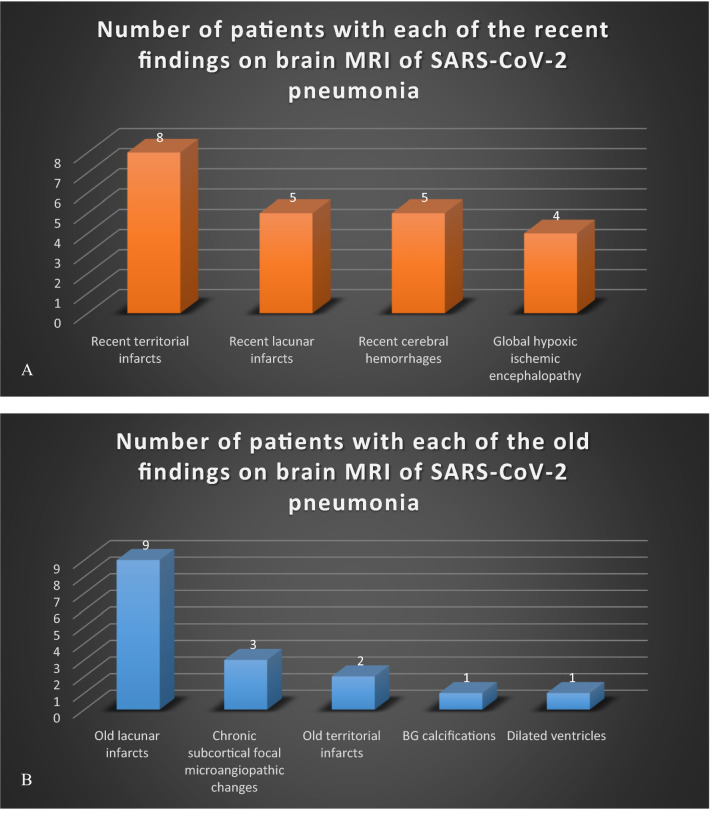
Frequency of patients with recent (**A**) and old (**B**) diagnostic findings on positive brain MRI of 38 among 46 patients with RT-PCR confirmed SARS-CoV-2 pneumonia and presented with neurological manifestations.

The finally selected 46 patients for analysis (33 females and 13 males) were aged 51.3 ± 18.4 years old. Thirty-six (78.3%) of them had comorbidities and 11 (23.9%) had > 3 comorbidities. Brain MRI revealed lesions in 38 (82.6%) patients and was negative in the remaining 8 (17.4%) patients of all finally enclosed patients with RT-PCR confirmed SARS-CoV-2 pneumonia. Brain MRI revealed recent lesions in 22 (57.9%) out of 38 patients with positive brain MRI studies (Fig. 2A). Out of such 22 patients with recent brain lesions, 17 (77.3%) were clinically significant, who represented (44.7%) of 38 patients with positive MRI and (37%) of all 46 RT-PCR confirmed SARS-CoV-2 pneumonia patients subjected to brain MRI. They comprised 13 patients with stroke: eight patients with recent infarcts and five patients with recent cerebral hemorrhage and four patients showed MRI findings of global hypoxic-ischemic encephalopathy (Fig. 2A). Out of 38 positive patients, MRI revealed old brain lesions in 16 (42.1%) positive patients (Fig. 2B).

The enclosed 46 patients were categorized into two groups: group A that comprised 29 (63%) patients and group B that comprised the remaining 17 (37%) patients, who were presented with focal neurological manifestations and non-focal neurological manifestations, at the time of brain MRI performance, respectively. Those age and gender-matched group A and group B patients (Table 1) showed negative brain MRI in seven patients and one patient, respectively. Interestingly, these groups expressed a strongly significant difference among them in Glasgow Coma Scale (GCS), but no significant difference in the relative set number of patient's comorbidities (Table1).

**Table 1:** Number of negative brain MRIs and characteristics of group A and B patients with RT-proved SARS-CoV-2 pneumonia, presented with neurological manifestations.

Presentation of neurological manifestations (symptoms and/or signs) candidate for brain MRI	Group A patients	Group B patients	*p *value
	Focal neurological manifestations	Non-focal neurological manifestations	
No. of patients; *n*	29	17	–
Age (years); (mean ± SD)	52 ± 15.3	52.1 ± 23	0.5**
Male gender; *n* (%)	21 (72%)	10 (59%)	0.2**
No. of comorbidities (mean ± SD)	2.1 ± 1.5	2.3 ± 1.8	0.4**
GCS (mean ± SD)	13.7 ± 3.2	8.7 ± 4.9	0.0006*
Negative brain MRI; *n* (%)	7 (24.1%)	1 (5.9%)	–
Old or chronic lesions on brain MRI; *n* (%)	8 (27.6%)	8 (47.1%)	–
Recent lesions on brain MRI; *n* (%)	14 (48.3%)	8 (47.1%)	–
Significant recent lesions on brain MRI; *n* (%)	11 (37.9%)	6 (35.3%)	–

*Significant correlation (*p* ≤ 0.05).

**No significant correlation (*p* > 0.05).

**Figure 3:**
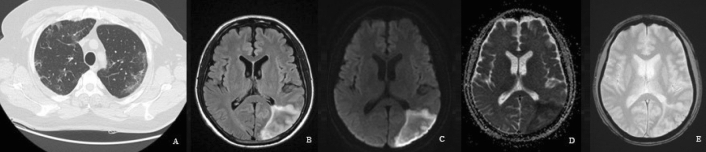
Fifty-eight-year-old male with RT-PCR proved SARS-CoV-2 pneumonia, (**A**) as revealed on chest CT. The patient also had right-sided weakness, right hemihyposthesia, and aphasia (stroke). Brain MRI was performed 4 days after the onset of the focal neurological manifestations as brain CT scanning was performed at the onset of such manifestations. (**B**) Axial FLAIR image showed increased signal intensity in the left parieto-occipital lobe (**C**) DW-MRI and (**D**) corresponding apparent diffusion coefficient (ADC) map showed diffusion restriction of the early sub-acute left parieto-occipital infarction. (**E**) No blooming was seen on two-dimensional (2D) T_2_* gradient-echo sequences (GRE), signifying no hemorrhage.

**Figure 4:**
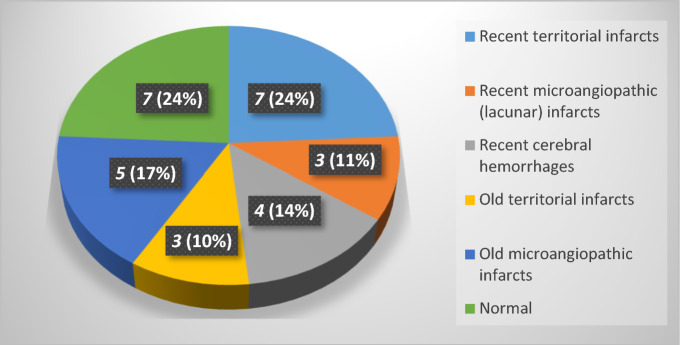
The distribution of patients, revealing brain MRI findings, in RT-PCR proved SARS-CoV-2 pneumonia, and presented with focal neurological manifestations.

Twenty-two patients of group A showed focal brain lesions on brain MRI: ten with recent infarcts; territorial infarcts in seven patients (Fig. 3) and lacunar or microangiopathic infarcts in three patients, four with recent cerebral hemorrhage, three with old territorial infarcts, and five patients found to have merely variable sized old lacunar infarcts (Fig. 4). The lesions on brain MRI of group B were 17 (37%) patients depicted in Table 2, which also showed group B patients' characteristics.

**Table 2:** Brain MRI findings in 17 patients, admitted with RT-PCR proved SARS-CoV-2 pneumonia, and presented with non-focal neurological manifestations.

Presentation	Impaired sensorium (8 patients)*	Post-cardiac arrest (5 patients)*	Headache (4 patients)*
Age (mean ± SD; years)	58.5 ± 18.1	38.2 ± 17.8	57.5 ± 34.5
Gender (female/male)	3/5	1/4	3/1
Comorbidities (mean ± SD)	2.75 ± 1.98	2.2 ± 1.79	1.25 ± 0.96
GCS (mean ± SD)	9.75 ± 3.8	5 ± 1.87	15 ± 0.93
No. of patients injected with contrast on brain MRI (female/male)	4/8	2/5	¾
Brain MRI findings	Recent territorial infarct: (1 patient)	Global hypoxic-ischemic encephalopathy: (4 patients)	LI –Recent LI: (1 patient) –Chronic LI: (1 patient)
	Cerebral hemorrhage: (1 patient)		Multiple bilateral cerebral focal subcortical lesions**: (2 patients)
	Lacunar Infarct (LI) –Recent LI: (1 patient) –Chronic LI: (3 patients)		Dilated ventricles: (1 patient)
	Basal ganglia (BG) calcification: (1 patient)		
	No abnormality: (1 patient)		

*Numbers in parentheses represents the number of patients within each subgroup.

**Details in Table 4 and Fig. 6.

**Figure 5:**
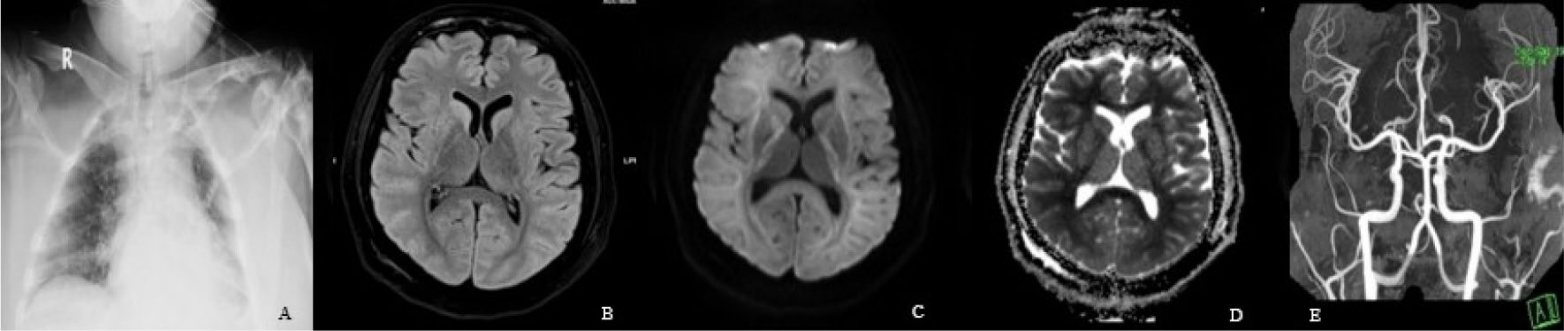
Twenty-six-year-old male with no prior co-morbidities; admitted with severe RT-PCR proved SARS-CoV-2 pneumonia, (**A**) as shown on the chest radiogram. This patient, who was on mechanical ventilation, went into cardiac arrest. Brain MRI was performed 4 days post arrest due to low GCS (= 6), revealed global hypoxic-ischemic encephalopathy. (**B**) Axial FLAIR image, at the level of the BG, showed decreased signal intensity in the thalami, while (**C**) corresponding DWI image showed lack of hyperintensity in both thalami, in addition to the caudate and putamen nuclei, findings that represent pseudo normalization. (**D**) Also, corresponding ADC map showed hyperintensity in the locations cited in (**B**). (**E**) Reconstructed maximum intensity projection magnetic resonance angiography (MRA) image shows no large vessel occlusion.

The brain MRI of four out of five (80%), among group B patients, who experienced cardiac arrest, showed a rather specific pattern, highly suggestive of global hypoxic-ischemic encephalopathy (Fig. 5). Such five patients were intubated, due to severe SARS-CoV-2 pneumonia, and had the lowest mean GCS among the group B patients (Table 2) and even among the whole patients (the latter mean ± SD of GCS = 11.8 ± 4.6). Brain MRI was performed 3–5 days after surviving the cardiac arrest, in all such patients. On considering those five patients, to be representing severe SARS-CoV-2 pneumonia (being intubated on mechanical ventilation), who experienced cardiac arrest and had low GCS, as group C (Table 3). The brain MRI of the remaining patients, who do not experience cardiac arrest and considered as group D, did not exhibit a single patient of global hypoxic-ischemic encephalopathy. Groups C and D were sex-matched; however, the mean age of group C was relatively younger (Table 3). As with group A and B categorization, no significant difference in the patient's number of comorbidities, but a highly significant difference in GCS (*p* = 0.0001) as presented in Table 3.

**Table 3:** The characteristics of the post-cardiac arrest patients, whose brain MRI showed global hypoxic-ischemic encephalopathy in four patients (80%) versus other patients with SARS-CoV-2 pneumonia.

Characteristics of the post-cardiac arrest patients	Post-cardiac arrest COVID-19 pneumonia (Group C)	Other COVID-19 pneumonia patients, not experiencing cardiac arrest (Group D)	*p* value
Age (mean ± SD)	38.2 ± 17.84	53.73 ± 17.77	0.04**
Female/male	1/4	14/27	0.53*
GCS (mean ± SD)	5 ± 1.9	13.24 ± 3.0	0.0001**
No. of comorbidities (mean ± SD)	2 ± 1.8	2.2 ± 1.6	0.94*
Contrast; female/male (%)	2/5 (40%)	26/41 (63.4%)	–

*Significant correlation (*p* ≤ 0.05).

**No significant correlation (*p* > 0.05).

**Figure 6:**
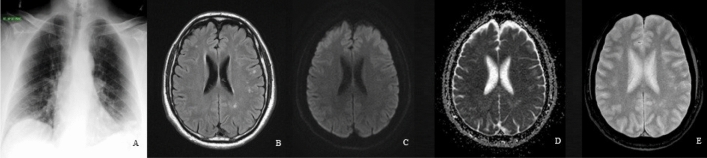
Thirty-three-year-old male, admitted with RT-proved SARS-CoV-2 pneumonia, (**A**) as noted on the chest radiogram. With GCS = 15, this patient developed headache and blurred vision. Brain MRI showed nonspecific scattered deep and sub-cortical small foci, in bilateral frontal and right parietal lobes with (**B**) hyperintense signals on axial FLAIR image. (**C**) DW-MRI, and (**D**) corresponding ADC map showed no diffusion restriction. (**E**) Also, no blooming was depicted on 2D T_2_* GRE image.

Interestingly, a pattern of bilateral scattered focal subcortical white matter lesions on brain MRI (hypointense on T_1_-WI and hyperintense on T_2_-WI and FLAIR images), suggestive of white matter microangiopathy, was solely revealed in two young-aged of group B patients (Fig. 6), was also demonstrated as an associated finding in three of group A patients, who were above 50 years old with recent and old lacunar infarcts. Among their patients' characteristics (Table 4), all had normal GCS.

**Table 4:** Characteristics of five admitted patients with SARS-CoV-2 pneumonia, whose brain MRI showed bilateral subcortical white matter focal lesions hypointense on T_1_WI and hyperintense on T_2_WI and FLAIR sequence MR images (Fig. 6).

Patients	Age (years)	sex	Co-morbidities and associated diseases	GCS	Contrast	Focal manifestations	Presentation	Associated brain MRI finding(s)
1	33	M	Alcoholism, HIV, and CMV	15	+	–	Headache	–
2	42	F	MERS-CoV, and PE	15	+	–	Headache	–
3	74	M	DM and CVA	15	–	+	Unsteady gait with unilateral weakness	Old LI
4	80	F	Hypo-thyroidism, HTN, DM, IHD, CVA, DVT, and PE	15	–	+	Left UMN facial muscles weakness	Old and recent LI
5	51	M	TIA	15	+	+	Left sided body weakness	Recent LI

Female *F*, Male *M*, Human immunodeficiency virus *HIV*, Cytomegalovirus *CMV*, Middle east respiratory syndrome-corona virus *MERS-CoV*, Pulmonary embolism *PE*, Diabetes mellitus *DM*, Cerebrovascular accident *CVA*, Deep vein thrombosis *DVT*, Upper motor neuron *UMN*, Transient ischemic attacks *TIA.*

## Discussion

All patients selected, with RT-PCR proved SARS-CoV-2 pneumonia, to be enclosed in our retrospective analysis, had neurological manifestations that may suggest brain involvement, and thence, subjected to brain MRI (Fig. 1). Desforges et al.^8^ stated that the increased frequency of neurological manifestations in patients with corona viral disease, including CNS manifestations, is concordant with the neurotropic nature of such virus. MRI revealed brain lesions in 38/46 (82.6%) of RT-PCR proved SARS-CoV-2 pneumonia patients, presented with neurological manifestations suggestive of CNS involvement and finally enclosed in this retrospective study. Recent brain lesions were shown in 22/38 (57.9%) of patients with brain MRI findings and 22/46 (47.8%) of enclosed patients. Even though, significant lesions represented only 17/46 (37%), most of them were recent territorial infarcts (8/46; 17.4%; Fig. 5) and cerebral hemorrhage (5/46; 10.9%). In the meta-analysis, by Nepal et al.^9^, ischemic stroke and intracerebral hemorrhage were among the CNS affection that uncommonly presented in SARS-CoV-2 infection.

Acute cerebrovascular diseases with infarcts were reported in overall 6/214 patients with COVID-19 infection (2.8%); among them, 5/88 with severe (5.7%) versus 1/126 with non-severe (0.8%) COVID-19 infection^4^. Also, upon retrospective cohort analysis, Merkler et al.^10^ found a relatively increased frequency of acute ischemic stroke in emergency and hospital amitted-COVID-19 patients (31/1916; 1.6%), compared to their corresponding ones with influenza (3/1486; 0.2%). In our study, the recent (acute and subacute) infarcts were found in eight patients (17.4%). Seven of those patients were presented with focal neurological manifestations and one only with impaired sensorium without localization. The relatively raised incidence of recent territorial infarcts, in our retrospective analysis, is due to the refined selection criteria of RT-PCR confirmed COVID-19 patients; as being admitted with clinicoradiologic evidence of pneumonia and presented with neurological manifestations suggesting CNS involvement and necessitating cross-sectional brain imaging.

Moreover, this study comprised both acute and subacute (recent) brain infarcts on MRIs, not only the acute ones, as reported in the meta-analysis. An Italian multicenter retrospective observational study reported 34/108 (31.5%) patients with ischemic strokes of hospitalized COVID-19 patients with acute neurological symptoms^11^. Almost near this latter frequency, Helmes et al.^3^ reported cerebral ischemic stroke, on MRI, in 3/13 patients (23%), among 58 observational consecutive patients admitted to the hospital because of acute respiratory distress syndrome (ARDS) due to RT-PCR proved-COVID-19. Nevertheless, Radmanesh et al.^5^ reported acute or subacute infarcts in 13 out of 242 RT-PCR proved SARS-CoV-2 patients (5.4%), who underwent brain cross-sectional imaging, as they had neurological manifestations suggestive of brain involvement. Again, the refined selection in our study, in addition to the age and gender heterogeneity among the patients enclosed in the comparative retrospective studies, are still the contributing factors for the relatively higher frequency of recent territorial infarcts in our patients. This prior debate can also be extrapolated to the higher relative percentage frequency of recent intracerebral hemorrhage in our study (5/46; 10.9%; Fig. 6) than that of acute intracerebral hemorrhage 11/242 (4.5%) patients reported by Radmanesh et al.^5^

Actually, four patients were among five patients presented with severe SARS-CoV-2 pneumonia, who experienced cardiac arrest, and their brain MRI showed a picture of global hypoxic-ischemic encephalopathy (Table 2). They represented 4/46 patients with SARS-CoV-2 pneumonia (8.7%) and 4/38 of them with positive brain MRI (10.5%). A retrospective study from China reviewed 274 patients of COVID-19, of which 24 (8.8%) developed hypoxic encephalopathy, which progressed to death in 23 (95.8%) and recovery in 1 (4.2%)^12^. As in our series, the global hypoxic-ischemic encephalopathy diagnoses, on MRI, in the series of Chen et al.^12^ were likely due to arrest-induced hypoxemia, rather than direct relation to SARS-CoV-2 pathogenesis. Among 242 proved COVID-19 patients, with neurological manifestations necessitating cross-sectional imaging, one patient had imaging findings of widespread anoxic brain injury following a large acute supra-and infratentorial hemorrhage^5^. In comatose cardiac arrest survivors (non-COVID-19 patients), the MR abnormalities were diffuse and extensive, involving the cortex, thalamus, and the cerebellum in patients who never awakened, meanwhile, the patients who awakened had normal or localized findings on MR images^13^.

Moreover, the leptomeninges in 28 patients, subjected to contrast-enhanced brain MRI among our selected patients, did not show contrast enhancement; two of them were of the post-arrest subgroup (group C) as shown in Table 3. However, Helmes et al.^3^ reported leptomeningeal enhancement in 8/13 patients, among their 58 observational series out of 64 consecutive patients admitted to the hospital because of acute respiratory distress syndrome (ARDS) due to RT-PCR proved COVID-19. Yet, only one (12.5%) of those eight patients, who presented with clinical neurologic features, had diffuse bi-frontal slowing of electroencephalogram (EEG) waves, consistent with encephalopathy and cerebrospinal fluid (CSF) RT-PCR for SARS-CoV-2 was negative in 7/7 (100%) of them^3^. Similarly, brain MRI was normal in two patients reports from USA and Italy, whose EEG revealed diffuse slowing consistent with encephalopathy^14^. Nevertheless, some researchers detected the nucleic acid in the cerebrospinal fluid of some patients and the brain tissue on autopsy of patients with other kinds of SARS-CoVs^15^.

The positive brain MRI in this study on confirmed patients of COVID-19 (SARS-CoV-2) pneumonia was relatively higher in the group A patients than those of group B; and as a logic finding was the relatively lower frequency of the MRI-revealed chronic brain lesions in-group A patients, as they presented with focal neurological manifestations (Table 1). Nevertheless, the percentage frequency of the recent and significant recent lesions on brain MRI were apparently almost equal in the two groups (Table 1). Interestingly also, there was no significant difference in the mean number of comorbidities between the two groups. The latter finding may indicate the predisposition of recent lesions by such factors. Actually, as stated before, four patients with brain MRI showed a picture of global hypoxic-ischemic encephalopathy were in-group B patients (Table 2). In addition, the MRI of two patients, with impaired sensorium without clinical localization, revealed recent cerebral infarct and recent cerebral hemorrhage, respectively. All such patients are responsible for the statistically highly significant lower GCS in-group B patients (Table 1), which showed relatively lesser negative brain MRIs than group A patients. In another term, relatively higher negative brain MRI were among the patients presented with focal neurological manifestations or relatively higher GCS (Table 1).

Nonspecific subcortical changes, suggestive of white matter microangiopathy, were found in five patients, on brain MRI, in our series (Table 3). Such lesions were the most common abnormal findings noted in 134/242 patients with SARS-CoV-2 presented with neurological manifestations in another series^5^. They found the lesions, to be as much as expected for age, in 108 (44.6%) patients; and more than expected for age in 26 patients (1%). In our series, two patients, below 50 years of age and presented with non-focal neurological manifestations, out of those aforementioned five patients, had such microangiopathic lesions as lone finding on brain MRI. Interestingly, they had laboratory evidence of prior viral infections, via serum IgG level assay and one of them got MERS-CoV infection (Table 3). Recombination of corona viruses, indicative of super infection with new corona virus is possible in the same patient, has been postulated^16^. The remaining three patients, who were above 50 years old and presented with focal neurological manifestations, had microangiopathic lesions, associated with lacunar infarcts on brain MRI. Nevertheless, all the five patients presented with the focal subcortical microangiopathic lesions on brain MRI in our series had full GCS (Table 3).

Despite the fact that the current study's strength is that it demonstrated the feasibility of brain MRI in SARS-CoV-2 pneumonia patients with newly developed neurological manifestations indicating brain involvement. However, because it is retrospective, has a small number of non-randomized patients (particularly group C), and there is no available baseline brain MRI study prior to SARS-CoV-2 infection, the current study may not accurately estimate the timing of some brain lesions and patterns on MRI. The age, gender, and comorbidity heterogeneity may limit the strong link between SARS-CoV-2 and cerebrovascular disease.

In conclusion, brain MRI is a feasible and important imaging modality in selected patients with SARS-CoV-2 pneumonia on developing neurological manifestations, suggestive of brain involvement, particularly in patients with non-focal manifestations and decline in GCS.

## Data Availability

The datasets generated during and/or analysed during the current study are available from the corresponding author on reasonable request.
